# Development of an Early Prediction Model for Subarachnoid Hemorrhage With Genetic and Signaling Pathway Analysis

**DOI:** 10.3389/fgene.2020.00391

**Published:** 2020-04-21

**Authors:** Wanjing Lei, Han Zeng, Hua Feng, Xufang Ru, Qiang Li, Ming Xiao, Huiru Zheng, Yujie Chen, Le Zhang

**Affiliations:** ^1^College of Computer Science, Sichuan University, Chengdu, China; ^2^College of Computer and Information Science, Southwest University, Chongqing, China; ^3^Department of Neurosurgery, Southwest Hospital, Third Military Medical University, Chongqing, China; ^4^State Key Laboratory of Trauma, Burn and Combined Injury, Third Military Medical University, Chongqing, China; ^5^School of Computing, Ulster University, Coleraine, United Kingdom

**Keywords:** bioinformatics, genomics, big data, artificial intelligence, genetics

## Abstract

Subarachnoid hemorrhage (SAH) is devastating disease with high mortality, high disability rate, and poor clinical prognosis. It has drawn great attentions in both basic and clinical medicine. Therefore, it is necessary to explore the therapeutic drugs and effective targets for early prediction of SAH. Firstly, we demonstrate that LCN2 can effectively intervene or treat SAH from the perspective of cell signaling pathway. Next, three potential genes that we explored have been validated by manually reviewed experimental evidences. Finally, we turn out that the SAH early ensemble learning predictive model performs better than the classical LR, SVM, and Naïve-Bayes models.

## Introduction

Subarachnoid hemorrhage (SAH) is the fastest developing and most critical hemorrhagic cerebrovascular disease, accounting for 5% of cerebrovascular diseases (Macdonald, 2014), and is associated with high rates of mortality and disability and poor clinical prognosis (Suarez et al., 2006). Although there have been significant advances in diagnostic methods, surgery, and endovascular techniques in recent years, the mortality rate of SAH remains as high as 15% (Macdonald et al., 2008).

Recent research has shown that early brain injury (EBI) may be the main cause of poor prognosis in SAH patients. Therefore, current SAH studies focus on exploring therapeutic drugs and targets for reduction of EBI after SAH and the early prediction of SAH (Sozen et al., 2011).

Lipocalin 2 (LCN2) is an acute secretory protein that regulates the pathophysiological processes of various organ systems in mammals and participates in the intrinsic immune protection of the central nervous system (CNS) (Flo et al., 2004; Ferreira et al., 2015). Studies of acute white matter injury in a mouse SAH model and the role of LCN2 in injury (Egashira et al., 2014) indicate that LCN2 plays an important part in SAH-induced white matter injury. Since above evidences suggest that LCN2 is closely related to SAH, we propose our first research question: is specific intervention for LCN2 (Warszawska et al., 2013) a promising SAH treatment strategy?

On the other hand, most previous studies (Chu et al., 2011; Ni et al., 2011; Zhang et al., 2017a) have only explored biomarkers for SAH prediction and treatment in a narrow molecular range, rather than taking a genome-wide approach. We propose our second research question: could we use a genome-wide approach to find potential biomarkers for SAH based on the effects of LCN2 treatment?

Previous studies have usually predicted SAH based on diagnostic imaging (Frontera et al., 2006; Ramos et al., 2019) and clinical automation data (Roederer et al., 2014), which may not provide enough predictive power. Thus, we propose our third research question: could we use key genes to build a more powerful early prediction model for SAH?

In this paper, we propose a new research plan to answer the above three research questions. First, we use SAH intervention experiments to screen out candidate genes that are susceptible to LCN2, then employ Fisher's exact test (Xie et al., 2011; Li et al., 2017; Xia et al., 2017; Zhang et al., 2019b) to choose signaling pathways from among the candidates under different experimental conditions. Second, we use E-Bayes (Carlin and Louis, 2010), SVM-RFE (Duan et al., 2005), SPCA (Zou et al., 2006), and statistical tests (Zhang et al., 2016, 2018, 2019b,d, 2020; Xiao et al., 2019) to investigate key genes from experimental data by considering both SAH and LCN2 as factors. Third, we integrate the logistic regression (LR), support vector machine (SVM), and Naive-Bayes algorithms (Xia et al., 2017; Zhang et al., 2017a, 2019a) into an ensemble learning model (Gao et al., 2017; Zhang et al., 2019b) to build a model for early SAH prediction.

First, manual review of the experimental evidence (Osuka et al., 2006; Majdalawieh et al., 2007; Hanafy et al., 2010; Hao et al., 2014; Kwon et al., 2015; Yu et al., 2018) demonstrates that we could intervene or treat SAH by targeting LCN2 from a cell signaling pathway perspective. Next, we explore three key genes that are sensitive to both SAH and LCN2 treatment, again using manual review of the experimental evidence (Huang et al., 2016; Sabo et al., 2017; Yu et al., 2018) to cross-validate the relationships between SAH and these key genes. Finally, we show that our SAH early prediction ensemble-learning model outperforms the classical LR, Naive-Bayes, and SVM models. In summary, we consider that this work provides a novel strategy for the future study of clinical treatment of SAH and related diseases.

## Materials and Methods

### Experimental Configuration

All experimental procedures were approved by the Ethics Committee of Southwest Hospital and were performed in accordance with the guidelines of the National Institutes of Health Guide for the Care and Use of Laboratory Animals.

#### Intervention Experiment for SAH

The original chip data for this experiment were provided by the Department of Neurosurgery, Southwest Hospital, PLA Military Medical University. SAH and sham-operated models were established; details are given in the Supplementary Material. Each experimental group included five mice, and the white matter area of the cerebral cortex was taken for gene chip testing. A total of 10 original chip samples were obtained from the SAH intervention experiments; these were divided equally into two groups as follows.

(1) SAH disease group: brain tissue in the white matter region of the cerebral cortex of SAH mice.

(2) Control group normal-1: brain tissue in the white matter region of the cerebral cortex of normal mice.

The chip was an Affymetrix GeneChip Mouse Gene 1.0 ST Array. Raw data included sample RNA extraction (white matter brain cells from the SAH model and from normal mice), sample RNA quality detection (total RNA>1 ug), cDNA synthesis, sense strand cDNA fragmentation, biotin labeling, chip hybridization, chip elution, and chip scanning. The raw data are available at http://www.ebi.ac.uk/arrayexpress/experiments/E-MTAB-8407.

We then carried out mass analysis and used the R Bioconductor package to perform quality control for each original chip (the SAH disease group and the control group normal-1). In the output gray scale image (Figure S1) for each chip sample, each chip name and the four corner patterns were very clear, and the contrast between light and dark was moderate.

**Figure 1:**
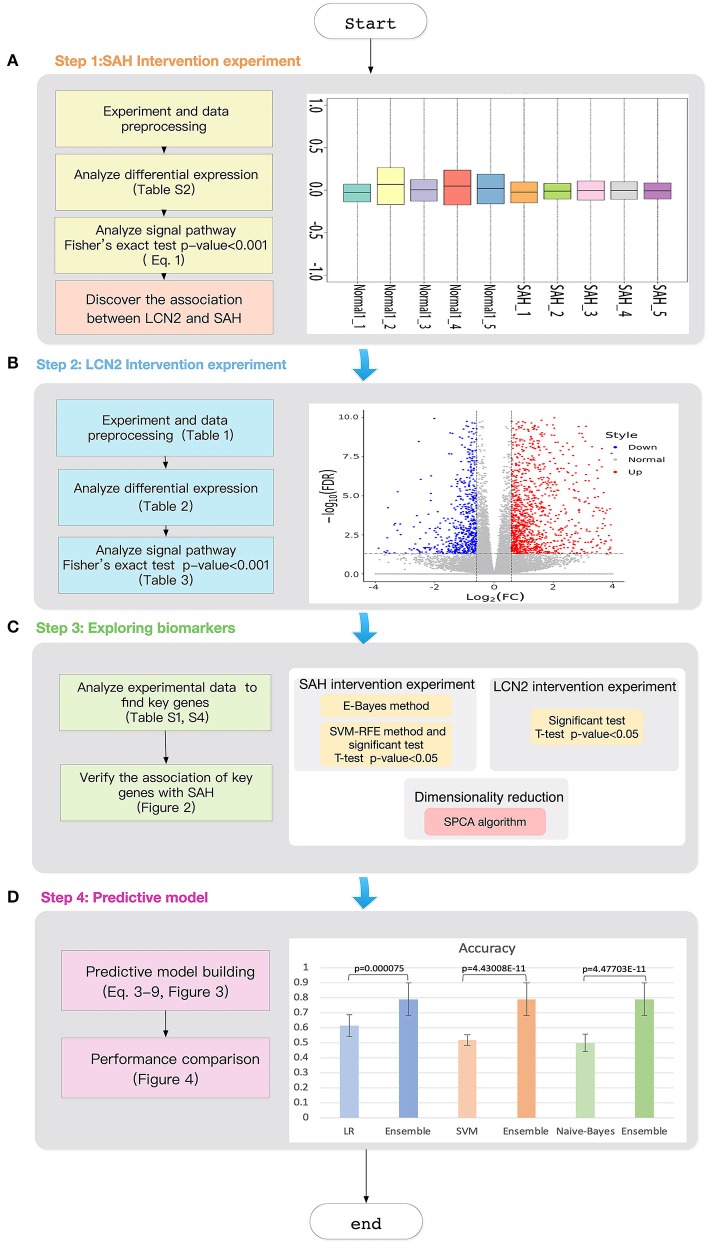
Workflow of the study. **(A)** SAH intervention experimental chip RLE box line diagram; the abscissa is log_2 (Median value of sample expression) and the ordinate represents each chip; **(B)** The volcano map of the comparison group SAH-siRNA-NC (1 day) vs normal-2. The abscissa is log_2_(*Fold change*) and the ordinate is −*log*_10_(*FDR*); The red point is the up-regulated gene, the blue point is the down-regulated gene, and the non-dispersive point is the non-differentiated gene; **(C)** Key gene screening workflow; **(D)** The accuracy for ensemble learning, LR, SVM and Naive-Bayes.

The right panel of Figure 1A shows the Relative Log Expression (RLE) boxplot for these 10 chips. The center of each sample was close to the position RLE = 0. This indicates that the expression levels of most genes in the sample were consistent. In addition, Figure S2 describes a normalized unscaled standard errors (NUSE) detection (Marta and Marc, 2014). Since Figure S2 shows that the center of each sample is close to the position NUSE = 1, we consider that the samples are too stable to have obvious batch effect. Then, we used Robust Multi-chip Analysis (RMA) (Irizarry et al., 2003) for data preprocessing, including background and perfect match probes (PM) correction, normalization, and summarization, to obtain the probe expression data matrix (Table S1). Finally, clustering analysis (Liu et al., 2019; Xiao et al., 2019; Zhang et al., 2019c; Wu and Zhang, 2020) (Figure S3) shows that the major differences between the chip of each group comes from SAH.

#### Intervention Experiment for LCN2

Here, in order to interfere with the expression of LCN2, 2 μL of specific short interfering RNAs (siRNAs) was delivered into the lateral ventricle with a Hamilton syringe. The injection was performed 48 h before SAH and three groups were used, as described below. We detail the procedures in the Supplementary Material.

(1) SAH-siRNA-LCN2: the SAH model was established and treated with intrathecal injection of LCN2 siRNA, and two samples were taken on the first and third days after surgery.

(2) SAH-siRNA-NC: the SAH model was established and treated with intrathecal NC siRNA, and two samples were taken on the first and third days after surgery, which helped us to remove the interference factors associated with the siRNA vector.

(3) Control group normal-2: the brain tissue of the white matter region of the cerebral cortex without any treatment.

The total number of samples in all experiments was 25 (Table 1). RNA sequencing was performed on the samples and the raw data are available at https://www.ncbi.nlm.nih.gov/sra/PRJNA575372.

**Table 1:** Experimental sample description after LCN2 intervention experiment.

**Sample**	**Number of samples**	**Description**
SAH-siRNA-LCN2(1day)	5	Mouse (SAH) brain cells, Intrathecal injection of LCN2 siRNA for 1 day
SAH-siRNA-LCN2(3day)	5	Mouse (SAH) brain cells, Intrathecal injection of LCN2 siRNA for 3 day
SAH-siRNA-NC(1day)	5	Mouse (SAH) brain cells, Intrathecal injection of blank siRNA for 1 day
SAH-siRNA-NC(3day)	5	Mouse (SAH) brain cells, Intrathecal injection of blank siRNA for 3 day
Normal-2	5	Mouse (normal) brain cells, blank control group-2

### Workflow of the Study

The workflow of the study is illustrated in Figure 1. First, we designed the intervention experiment for SAH detailed in section “Intervention Experiment for SAH”, which allowed us to obtain the differential genes under different experimental conditions. Based on these differential genes, we could identify the key signaling pathways.

As targeting LCN2 could result in changes in these related signaling pathways (causing remission or promotion of SAH), we consider that LCN2 plays an important part in the entire biological cell process for SAH.

Next, we used an intervention experiment for LCN2 to obtain gene expression levels for diseased and normal mouse brain cells at different time points. Then, we employed commonly used dimensional reduction algorithms to explore three key genes under the impact of both SAH and LCN2 treatment.

Finally, we used these three key genes as classifiers to develop an ensemble learning model for early SAH prediction, the predictive power of which was much better than that of the classic LR, Naive-Bayes, and SVM models.

## Results

### Signaling Pathway Analysis

#### Differentially Expressed Gene Selection

We used E-Bayes, one of the most commonly used methods for differential expression analysis (Edwards et al., 2005), to screen the differential genes by setting *Fold change* ≥ 1.5 and *p*-value < 0.05. Table S2 lists 2942 differentially expressed genes, accounting for 10.16% of the total number of genes (28,944). Among them, there were 1016 and 1926 genes with upregulated and downregulated expression (Figure S4), respectively.

#### Pathway Analysis

We used Equation 1 and the data in Table S3 to explore related signaling pathways by carrying out Fisher's exact test (Xia et al., 2017) using Kobas 3.0 (Wu et al., 2006; Xie et al., 2011; Ai and Kong, 2018) for the differentially expressed genes from Table S2.

(1) $$\begin{array}{l} p_{F}\left(n_{f},n,N_{f},N\right)=2\;*\;\overset{n_{f}}{\underset{x=1}{\sum }}\frac{\left(\begin{array}{c} n\\ x \end{array}\right)\left(\begin{array}{c} N-n\\ N_{f}-x \end{array}\right)}{\left(\begin{array}{c} N\\ N_{f}\\ \; \end{array}\right)} \end{array}$$

Here, *N* is the number of genes in the sample and *n* is the number of genes contained in the pathway. *N*_*f*_ is the number of differentially expressed genes and *n*_*f*_ is the number of differentially expressed genes included in the pathway.

The Fisher's exact test assumes *H*_0_:*p*_1_ = *p*_2_; the alternative hypothesis is *H*_1_:*p*_1_ ≠ *p*_2_. *p*_1_is the probability that the differentially expressed gene will fall in the pathway, and *p*_2_ is the probability that the non-differentiated gene does not fall in the pathway. The p-value (*p*_*F*_) of Fisher's exact test was obtained by Equation 1.

Table S2 lists 70 signaling pathways for which the p-value was less than 0.001. LCN2 is a protein involved in MAPK signaling pathways that protects the CNS as part of the innate immune system (Warszawska et al., 2013). Previous studies have shown that LCN2 activates phosphorylation of p38 MAPK, which phosphorylates the Ser168 and Ser170 sites of NFATc4 and inhibits nuclear translocation of NFATc4 (Olabisi et al., 2008). NFATc4 is a key factor in remyelination and closely related to SAH, indicating that white matter damage after SAH is associated with remyelination (Kao et al., 2009; Guo et al., 2017).

Therefore, we hypothesize that LCN2 could promote the phosphorylation of transcription factor NFATc4 and inhibit its nuclear transcription by activating p38 MAPK, thereby preventing remyelination and causing white matter damage after SAH.

#### LCN2 Intervention Experimental Results Analysis

To prove our hypothesis, we designed a LCN2 intervention experiment (Figure 1B) to test whether LCN2 could affect SAH from the perspective of the differential expressed genes and the related signaling pathways.

First, we used the DESeq2 (Varet et al., 2016) method to select differentially expressed genes from SAH-siRNA-LCN2 and normal-2, SAH-siRNA-NC and normal-2, and SAH-siRNA-LCN2 and SAH-siRNA-NC groups on days 1 and 3, respectively (Table 1). The results are shown in Table 2, Table S4, and Figure S5.

**Table 2:** Differential expressed genes for different experimental group.

**Experimental group**	**Total number of genes**	**Up-regulation of genes**	**Down-regulation of genes**
SAH-siRNA-LCN2(1day) VS normal-2	25342	1541	634
SAH-siRNA-LCN2 (3day) VS normal-2	25055	1264	451
SAH-siRNA-NC(1day) VS normal-2	25384	1159	556
SAH-siRNA-NC(3day) VS normal-2	25564	1297	409
SAH-siRNA- LCN2 (1day) VS SAH-siRNA-NC(1day)	25293	99	14
SAH-siRNA- LCN2 (3day) VS SAH-siRNA-NC(3day)	25251	5	18

Next, we used Kobas 3.0 (Wu et al., 2006; Xie et al., 2011; Ai and Kong, 2018) to carry out Fisher's exact test for the differential genes in Table 2, to identify related signaling pathways (Table S5). Next, we used the manually reviewed evidence (Osuka et al., 2006; Majdalawieh et al., 2007; Hanafy et al., 2010; Hao et al., 2014; Kwon et al., 2015; Yu et al., 2018) to cross-validate the SAH-related signaling pathways in Table S5. Table 3 lists the cross-validated SAH-related signaling pathways.

**Table 3:** Cross-validated SAH related signaling pathway.

**Experimental group**	**Important pathways related to SAH**
SAH-siRNA-LCN2 (1day) VS normal-2	PI3K-Akt (Hao et al., 2014), Jak-STAT (Osuka et al., 2006), p53 (Yu et al., 2018), TNF (Hanafy et al., 2010), Toll-like receptor (Kwon et al., 2015), NF-kappaβ (Majdalawieh et al., 2007)
SAH-siRNA-LCN2 (3day) VS normal-2	PI3K-Akt (Hao et al., 2014), Jak-STAT (Osuka et al., 2006), p53 (Yu et al., 2018), TNF (Hanafy et al., 2010), Toll-like receptor (Kwon et al., 2015), NF-kappaβ (Majdalawieh et al., 2007)
SAH-siRNA-NC (1day) VS normal-2	PI3K-Akt (Hao et al., 2014), Jak-STAT (Osuka et al., 2006), TNF (Hanafy et al., 2010), Toll-like receptor (Kwon et al., 2015), NF-kappaβ (Majdalawieh et al., 2007)
SAH-siRNA-NC (3day) VS normal-2	PI3K-Akt (Hao et al., 2014), Jak-STAT (Osuka et al., 2006), TNF (Hanafy et al., 2010), Toll-like receptor (Kwon et al., 2015), NF-kappaβ (Majdalawieh et al., 2007)
SAH-siRNA- LCN2 (1day) VS SAH-siRNA-NC (1day)	TNF (Hanafy et al., 2010), Toll-like receptor (Kwon et al., 2015)
SAH-siRNA- LCN2 (3day) VS SAH-siRNA-NC (3day)	Transcriptional misregulation in cancer (Lee and Young, 2013)

As shown in Table 3, all the experimental groups had SAH-related signaling pathways except the transcriptional misregulation in cancer signaling pathway (Lee and Young, 2013) in the SAH-siRNA-LCN2 (3 day) vs. SAH-siRNA-NC (3 day) experimental group. However, as one of the proteins from this pathway, Gzmb (Table S5), is closely associated with post-ischemic brain cell death (Chaitanya et al., 2010), we consider that it could be a new target for secondary brain injury inhibition (Armstrong et al., 2017). Therefore, we conclude that specific intervention for LCN2 is a promising SAH treatment strategy.

### Feature Selection

After demonstrating the impact of LCN2 on SAH, we chose potential biomarkers for SAH using a genome-wide approach. Figure 1C shows the workflow used to choose key genes that were not only related to both SAH and LCN2 but were also insensitive to treatment at different time points. Figure 1C shows the following three modules.

(1) SAH intervention experiment module

Owing to the large number of differential genes (Table S2), it was necessary to further narrow down the scope of the screening. First, we used the E-Bayes method (Edwards et al., 2005) to filter the probe expression data matrix (Table S1) by the E-Bayes function of R's limma package (Smyth et al., 2005). The differential probes were obtained by setting the filter parameters to *Fold change* ≥2 and *p*-value < 0.05.

Second, we used SVM-RFE (Duan et al., 2005) (Equation 2) to rank the genes in the probe expression data matrix, and then carried out the *t*-test and *F*-test (Zhang et al., 2017b) for the top 100 genes.

(2) $$\begin{array}{l} \left\{ \begin{array}{c} DJ\left(i\right)=\left(1/2\right)\alpha^{T}H\alpha -\left(1/2\right)\alpha^{T}H\left(-i\right)\alpha \\ H=y_{i}y_{j}K\left(x_{i},x_{j}\right) \end{array}\right. \end{array}$$

where *y*_*i*_ and *y*_*j*_ represent the classification labels of probes *x*_*i*_ and *x*_*j*_, respectively; *K*(*x*_*i*_, *x*_*j*_) is the kernel function, *i, j* = 1, 2, …, *n*; α is obtained by training the SVM classifier; *DJ*(*i*) is the sort function; and *H* is the matrix.

We then combined the results of these two methods to obtain the significant probes for both the E-Bayes and SVM-RFE methods.

Finally, we used the transcription cluster annotation file (version: MoGene-1_0-st-v1) downloaded from the Affy (Gautier et al., 2004) website to extract the gene ID for these probes, resulting in 47 key genes (Table S6).

(2) LCN2 intervention experiment module

We performed *t*-tests and *F*-tests (Zhang et al., 2017b) for the key genes (Table S6) in the SAH-siRNA-LCN2 (1 day) vs. normal-2 and SAH siRNA-LCN2 (3 day) vs. normal-2 groups (Table S4).

There were 15 and 13 statistically significantly differential genes for the SAH-siRNA-LCN2 (1 day) vs. normal-2 group (Table S7) and the SAH-siRNA-LCN2 (3 day) vs. normal-2 group (Table S8), respectively. Taking the intersection of the results from these two experimental groups gave nine key genes, Tk1, Cyr61, Nupr1, Dcn, Lum, Olig1, Pcolce2, Slc6a9, and Kcnt2, which were sensitive to both SAH and LCN2 intervention, regardless of treatment, at different time points.

(3) Dimensional reduction module

**Figure 2:**
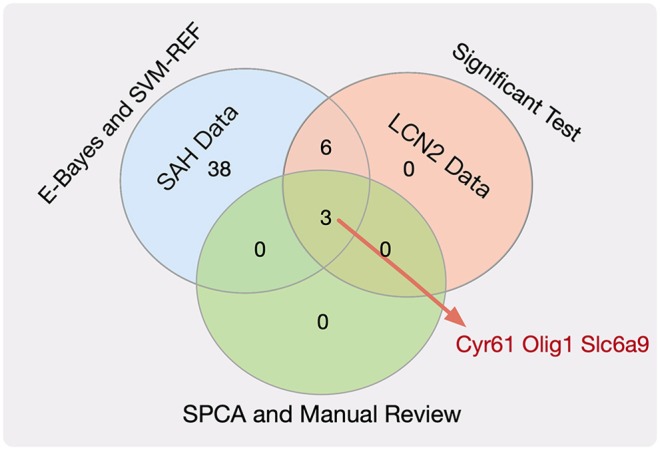
Venn plot for the key genes.

Next, we employed the SPCA algorithm (Zou et al., 2006; Li et al., 2017) to perform dimensional reduction for the nine key genes. This resulted in five candidate genes (Tk1, Cyr61, Olig1, Slc6a9, and Pcolce2). However, manual review of the experimental evidence indicated that only Cyr61 (Yu et al., 2018), Olig1 (Sabo et al., 2017), and Slc6a9 (Huang et al., 2016) were closely related to SAH, cerebral hemorrhage, and brain injury. Therefore, we considered these three genes (Figure 2, Table S9) to be potential biomarkers for SAH.

### Ensemble Learning Model

#### Early SAH Prediction Model

This study used three classification algorithms, LR (Hosmer et al., 2013), SVM (Suykens and Vandewalle, 1999), and Naive-Bayes (Wang et al., 2007) to develop the SAH prediction model, using the selected key genes as the respective classifiers. These three classic methods were then integrated into a novel ensemble learning model to improve the predictive accuracy.

**Figure 3:**
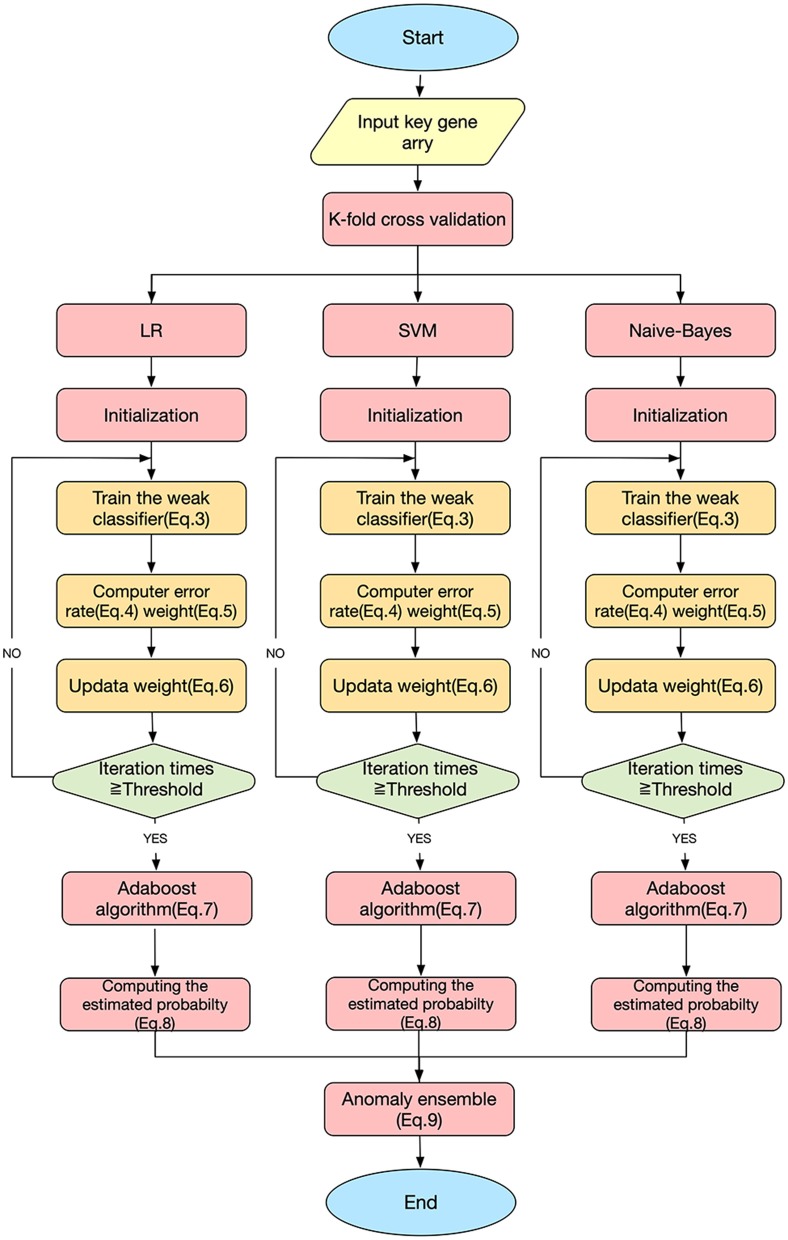
SAH predictive ensemble learning model.

Figure 3 shows the workflow of the SAH prediction model, based on our previous studies (Li et al., 2017; Xia et al., 2017; Zhang et al., 2019b). The key equations of the model are as follows.

(3) $$\begin{array}{l} D_{t}\left(i\right)=\frac{1}{n} \end{array}$$

(4) $$\begin{array}{l} \varepsilon_{t}=\frac{number\;of\;incorrectly\;classified\;samples}{total\;number\;of\;samples} \end{array}$$

(5) $$\begin{array}{l} \alpha_{t}=\frac{1}{2}\text{ln}\frac{1-\varepsilon_{t}}{\varepsilon_{t}} \end{array}$$

(6) $$\begin{array}{l} D_{t+1}\left(i\right)=\frac{D_{t}\left(i\right)}{sum\left(D\right)}\left\{ \begin{array}{c} \text{exp}\left(-\alpha_{t}\right),\;if\;h_{t}\left(x_{i}\right)=y_{i}\\ \text{exp}\left(\alpha_{t}\right),\;if\;h_{t}\;\left(x_{i}\right)\neq y_{i} \end{array}\right. \end{array}$$

(7) $$\begin{array}{l} H_{m}\left(x\right)=sign\overset{T}{\underset{t=0}{\sum }}\alpha_{t}h_{t}\left(x\right) \end{array}$$

(8) $$\begin{array}{l} E_{H_{m,}}=\overset{3}{\underset{m=1}{\sum }}P_{H_{m}} \end{array}$$

(9) $$\begin{array}{l} Y\left(x\right)=\left\{ \begin{array}{c} 1\;E_{H_{m}}\geq 0.5\\ 0\;E_{H_{m}}<0.5 \end{array}\right. \end{array}$$

Here, *D*_*t*_(*i*) is the weight distribution, *t* is the iteration time, *i* is the index of the sample, and *n* is the number of the sample. ε_*t*_ and α_*t*_ are the error rate and weight of each weak classifier *h*_*t*_, respectively. For a sample set *S* = { (*x*_1_, *y*_1_), (*x*_2_, *y*_2_), …, (*x*_*n*_, *y*_*n*_) }, *x*_*n*_ are the samples and *y*_*n*_ ∈ {0, 1} are the labels; *y*_*i*_=0 indicates that *x*_*i*_ is not an SAH patient, and *y*_*i*_=1 indicates that *x*_*i*_ is an SAH patient. *H*_*m*_ is the homomorphic integration for each weak classifier *h*_*t*_; m is the index of the weak classifier, m = 1, 2, 3; *T* is the threshold of the iteration time; *P*_*H*_*m*__ is the predictive probability of disease; and *E*_*H*_*m*__is the estimated probability of the model *H*_*m*_. *Y* (*x*) is the result of the final classifier obtained by a voting method (Dietterich, 2000).

#### Predictive Performance Comparison

**Figure 4:**
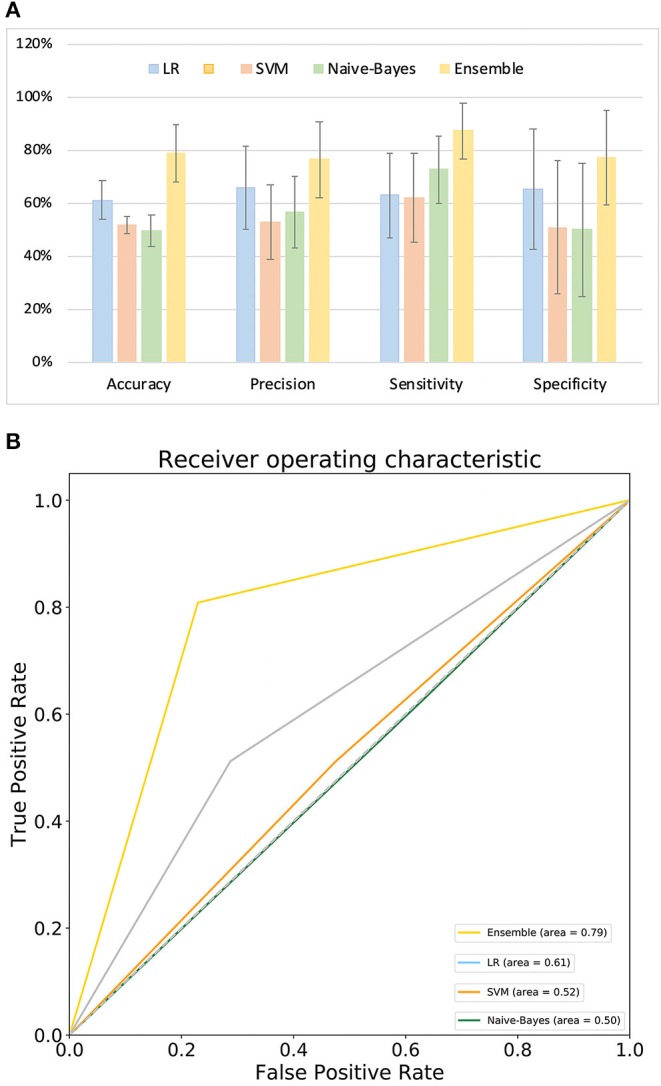
Model performance. **(A)** Comparison of classification performance of LR, SVM, Naive-Bayes, and ensemble learning model; **(B)** ROC chart plotted for LR, SVM, Naive-Bayes, and ensemble learning model.

Figure 4A compares the classification performance for the LR, Naive-Bayes, SVM, and ensemble learning models, based on four commonly used classification measurements (Table S10) (Zhang et al., 2019b). The numerical values used in Figure 4A are listed in Table S11; these demonstrate that the ensemble learning method outperforms the other three methods with respect to accuracy, precision, sensitivity and specificity. The ROC chart plotted in Figure 4B compares the classification effects of LR, Naive-Bayes, SVM, and ensemble learning models. The classification effect of ensemble learning models is also superior to the other three.

## Discussion

This study aimed to interrogate the potential therapeutic targets of SAH and use them as classifiers to develop a model for early prediction of SAH.

To achieve this aim, we proposed the following three scientific questions. First, is specific intervention involving LCN2 a promising SAH treatment strategy? Second, could we choose potential biomarkers for SAH at a genome-wide level by considering the effects of LCN2? Third, could we use key genes to build an SAH early prediction model with strong predictive power?

Regarding the first question, as the manually reviewed experimental evidence (Osuka et al., 2006; Majdalawieh et al., 2007; Hanafy et al., 2010; Hao et al., 2014; Kwon et al., 2015; Yu et al., 2018) and the results in Table 3 all indicate that LCN2-related signaling pathways play an important part in the pathogenesis SAH, we propose that LCN2 could promote or alleviate SAH-related diseases, and could also be used to treat SAH in the future.

To answer the second question, we used mathematical algorithms to explore five potential gene biomarkers (Tk1, Cyr61, Olig1, Slc6a9, and Pcolce2), considering the impact of both SAH and LCN2 treatment at different time points, and also used the manually reviewed experimental evidence to demonstrate that Cyr61 (Yu et al., 2018), Olig1 (Sabo et al., 2017), and Slc6a9 (Huang et al., 2016) were closely related to SAH. Although Tk1 and Pcolce2 have not been reported to be associated with SAH, we will investigate their connections in future work.

Regarding the third question, although this study represents significant progress in SAH prediction, it had several drawbacks. For example, the SAH intervention experiment sample size was too small for us to demonstrate high predictive accuracy for the model. In future work, we will integrate more recent bioinformatics research algorithms (Zhang et al., 2016, 2017a, 2018, 2019a,d; Gao et al., 2017; Zhang and Zhang, 2017) and data into the system to overcome the problems.

In summary, this study analyzed the impact of LCN2 on SAH and explored the key biomarkers of SAH under LCN2 treatment at different time points. An ensemble learning model was developed to predict SAH occurrence. The results demonstrate that LCN2 (Warszawska et al., 2013) can effectively intervene in or treat SAH from a cell signaling pathway perspective. Also, three key genes were identified and validated by manual review of the experimental evidence (Huang et al., 2016; Sabo et al., 2017; Yu et al., 2018). Finally, the results showed that the ensemble learning model performed better for early SAH prediction than the classical LR, SVM, and Naive-Bayes models.

## Data Availability Statement

The raw data supporting the results of this article can be found in ArrayExpress (accession ID: E-MTAB-8407) and BioProject (accession ID: PRJNA575372).

## Ethics Statement

The animal study was reviewed and approved by the Ethics Committee of Southwest Hospital.

## Author Contributions

LZ and YC conceived the study and developed the model. HZe and WL performed the simulations for the model. WL and HZe wrote the manuscript. MX and HZh performed the analysis for the model. HF, XR, and QL contributed to acquisition of data. All authors read and approved the final manuscript.

## Conflict of Interest

The authors declare that the research was conducted in the absence of any commercial or financial relationships that could be construed as a potential conflict of interest.
