# Supplementary material for: Development of an Early Prediction Model for Subarachnoid Hemorrhage With Genetic and Signaling Pathway Analysis
Source: Front Genet. 2020 Apr 21;11:391. doi: 10.3389/fgene.2020.00391 (PMC7186496; doi:10.3389/fgene.2020.00391)
Supplement: Supplementary file 1 [file Data_Sheet_1.pdf]

Supplementary Material

1 Supplementary Figures and Tables

1.1 Supplementary Figures

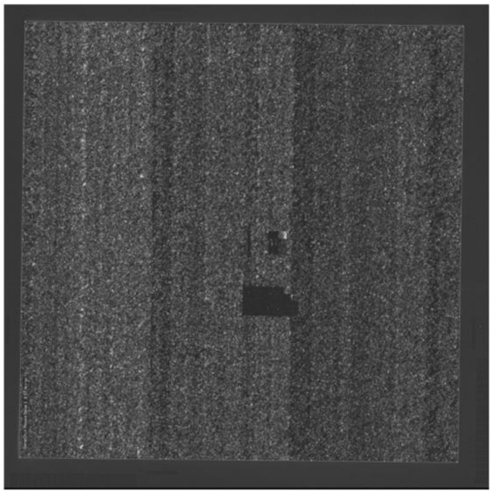

(A) SAH_1

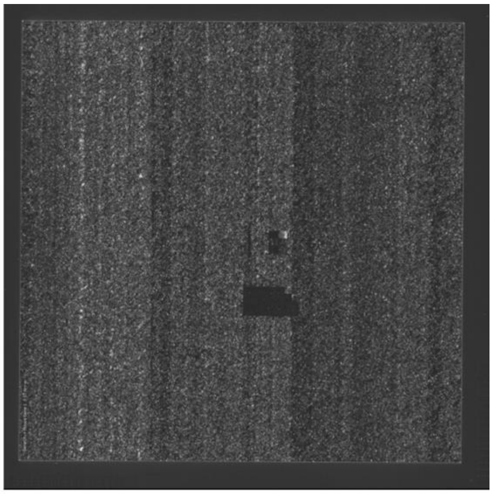

(B) SAH_2

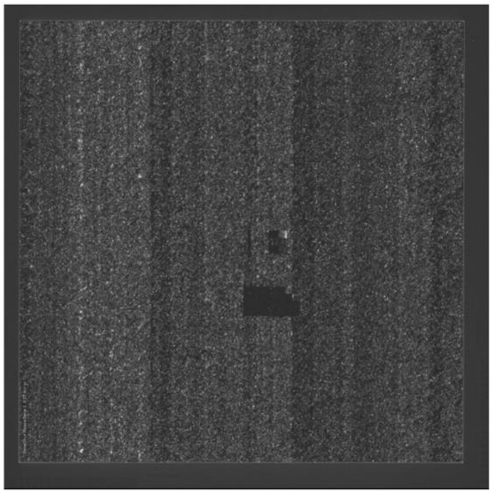

(C) SAH_3

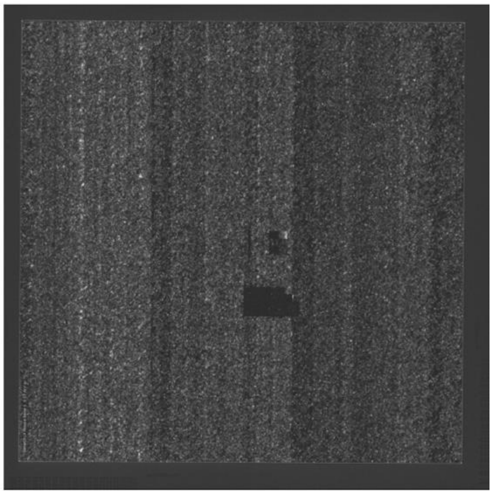

(D) SAH_4

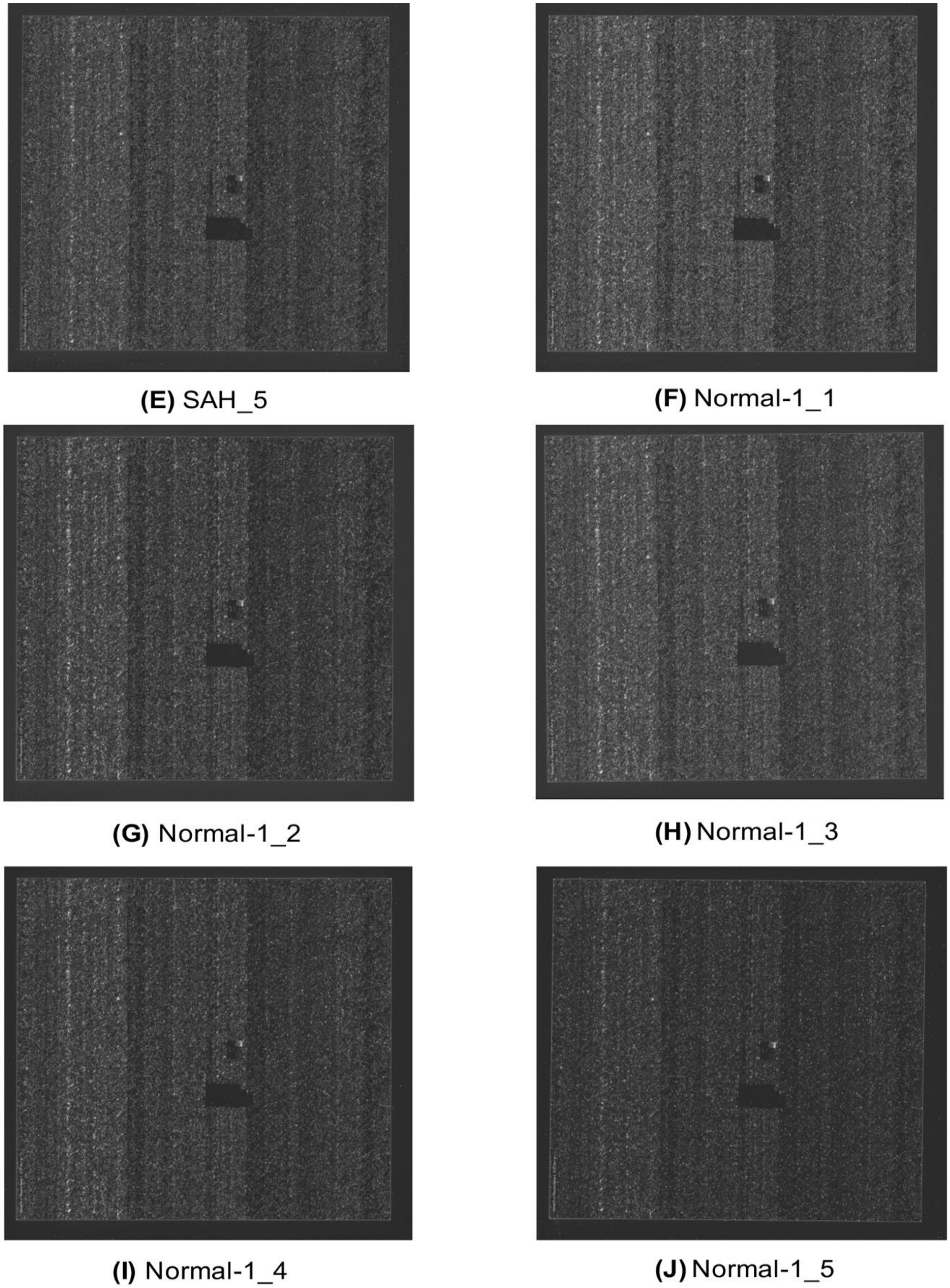

Figure S1. Gray scale image for SAH intervention experiment. Ten original chip gray scale images of SAH intervention experiment; **(A)-(E)** are the gray scale images for five experimental group chips; **(F)-(J)** are the gray scale images for five control group chips.

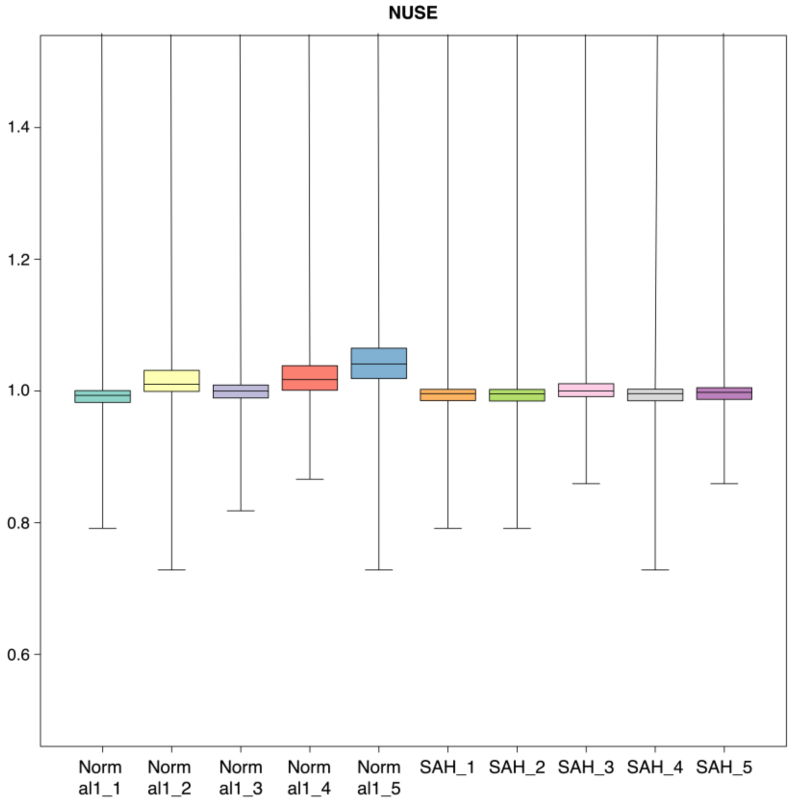

Figure S2. SAH intervention experimental chip NUSE boxplot.

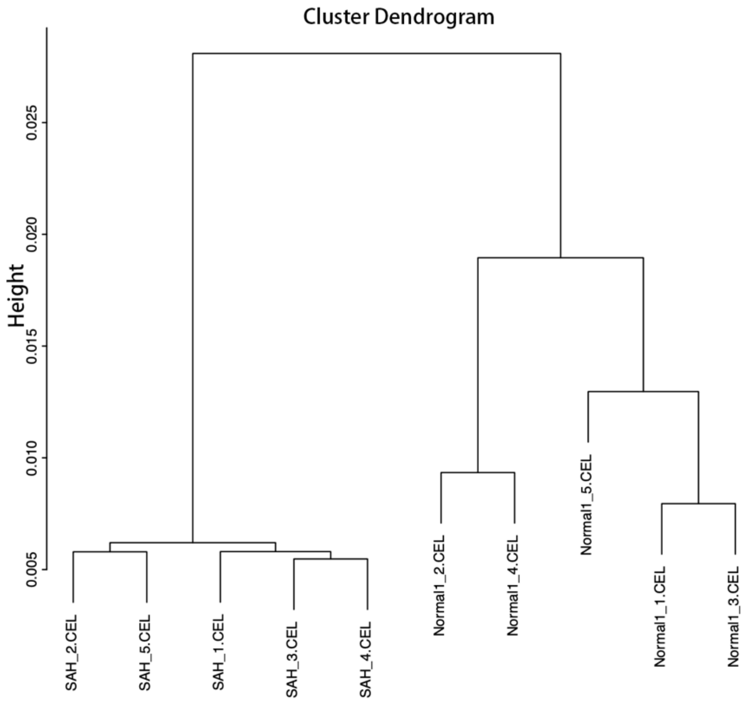

Figure S3. Cluster graph for SAH intervention experiment.

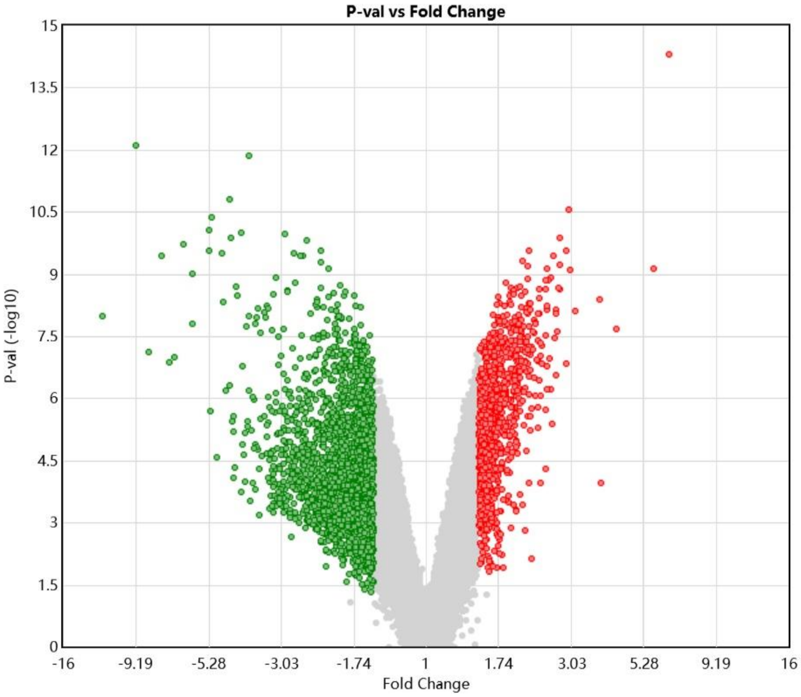

Figure S4. Volcanic maps for SAH intervention experiment. The volcano map of the comparison group SAH vs normal-1. The abscissa is $log_2(Fold\ change)$ and the ordinate is $-log_{10}(FDR)$. The red, green and non-dispersive points represent the up-regulated, down-regulated, and non-differentiated gene, respectively.

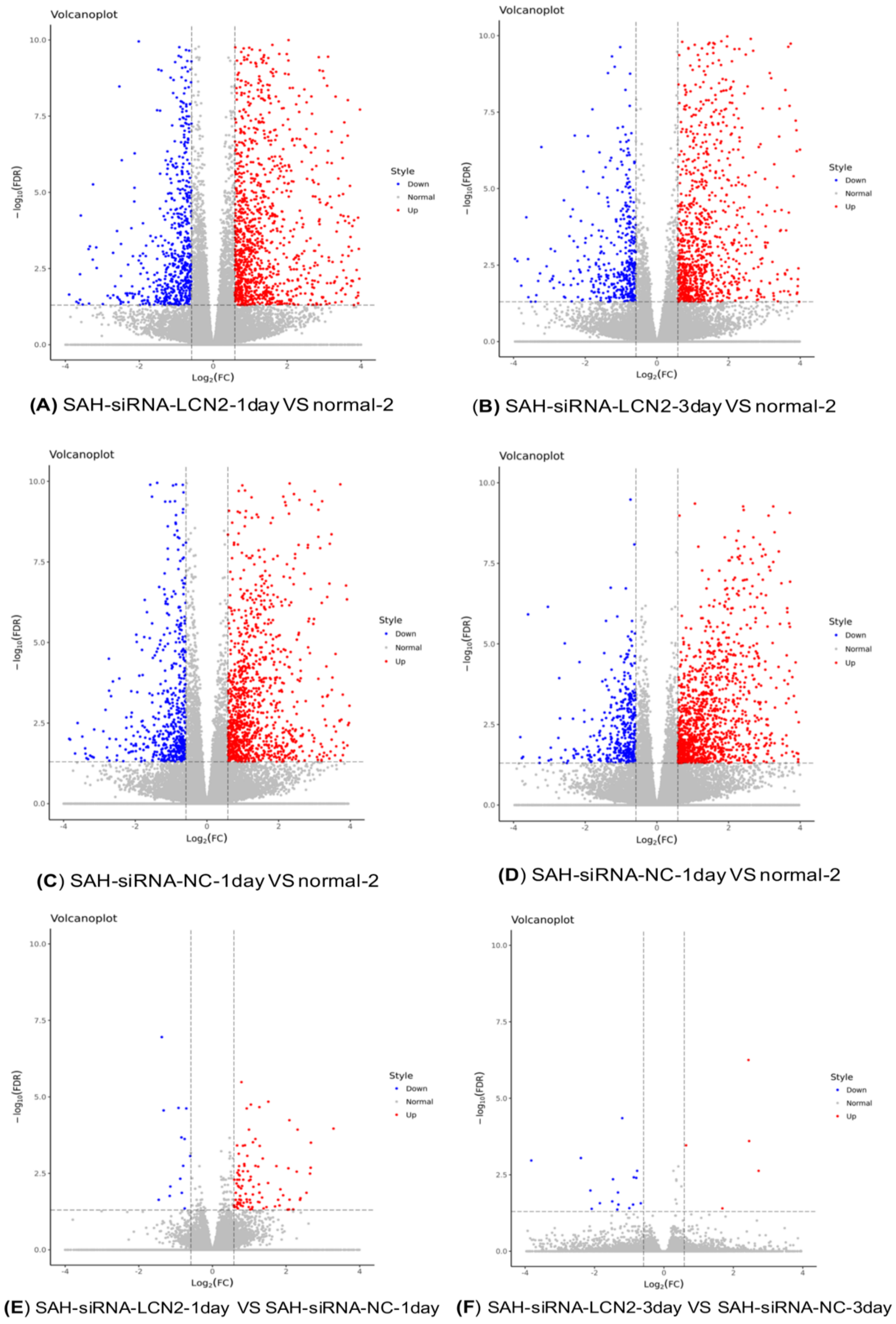

Figure S5. Volcanic maps for LCN2 intervention experiment. The volcano map of the different group. The abscissa is $log_2(Fold\ change)$ and the ordinate is $-log_{10}(FDR)$. The red, blue and non-dispersive points represent the up-regulated, down-regulated, and non-differentiated gene, respectively.

1.2 Supplementary Tables

Table S1. Probes expression data matrix.

The experimental probe expression matrix for each group in the SAH intervention experiment.

Table S2. SAH intervention experiment analysis results.

The results of differentially expressed gene analysis and related signaling pathway analysis in SAH intervention experiments.

Table S3. Fisher's exact Test for the signaling pathway.

	Differentially expressed gene	Non-differentiated gene	Total
Included in pathway	n_f	$n - n_f$	n
Not included in pathway	$N_f - n_f$	$(N - n_f) - (n - n_f)$	$N - n$
Total	N_f	$N - N_f$	N

Table S4. Differential genetic analysis results for LCN2 interventional experiments.

Results of differential genetic analysis for each group of data in the LCN2 intervention experiment.

Table S5. Signaling pathway analysis results for LCN2 intervention experiments.

Results of signaling pathway analysis of each group of data in the LCN2 intervention experiment.

TableS1, S2, S4 and S5 are available on https://github.com/charlotte5683/supplementary-of-SAH.git.

Table S6. 47 key genes for both e-Bayes and SVM-RFE methods.

**Gene name**

Cyb5r1	Pcolce2
Kcnt2	Tagln
Ddr2	mt-Tr
Igf1	mt-Ts2
Lum	Slc7a3
Dusp6	Capn6
Tk1	Tnfsf18
Gm24564	Gm39701
Pck2	Moxd1
Tmem74	Dcn
Zfp942	Aldh1l2
Ttr	Meg3
Stk32a	Ero1l
Chac1	Enpp2
Trib3	Mir99ahg
Postn	Olig1
Slc7a11	Ankrd12
Cyr61	Acta2
Slc6a9	Fbln7
Akap9	P2rx3
Mir344-2	Cth

Trim66	Gabra2
Nupr1	Cyb5r2
Ednra	

Table S7. Significantly differential genes for SAH-siRNA-LCN2(1day) VS normal-2 group.

**SAH-siRNA-LCN2(1day) VS normal-2 group**	
Olig1	Pck2
Cyb5r1	Kcnt2
Tk1	Nupr1
Dcn	Lum
Ednra	Pcolce2
Slc6a9	Slc7a11
Cyr61	Trib3
Akap9	

Table S8. Significantly differential genes for SAH-siRNA-LCN2(3day) VS normal-2 group.

**SAH-siRNA-LCN2(3day) VS normal-2 group**	
Tk1	Slc6a9
Cyr61	Dusp6
Aldh1l2	Olig1

Nupr1	Igf1
Dcn	Kcnt2
Lum	
Pcolce2	
Tnfsf18	

Table S9. Input samples for the prediction model.

Gene Name	SAH_1	SAH_2	SAH_3	SAH_4	SAH_5
**Cyr61**	5.8942628	5.96110867	5.93479255	6.02621404	5.73609078
**Olig1**	7.0429023	7.56910104	7.21385307	7.27041593	7.43777252
**Slc6a9**	9.4912445	9.20928367	9.54588542	9.43792448	9.01581096
**Gene Name**	**Normal-1_1**	**Normal-1_2**	**Normal-1_3**	**Normal-1_4**	**Normal-1_5**
**Cyr61**	7.3059794	7.56463002	7.19854145	7.74025933	7.38489239
**Olig1**	8.8771356	9.18547761	8.8943184	9.011647	8.88975631
**Slc6a9**	7.1622279	7.28458596	7.34258084	7.42990633	7.09533177

Table S10. Model performance indicator.

Index	Formula	Illustration
**Accuracy**	$\frac{TP+TN}{P+N}$	TP: actual illness and is recognized as disease
**Precision**	$\frac{TP}{TP+FP}$	TN: not actually diseased and is recognized as a disease

**Sensitivity**	$\frac{TP}{TP+FN}$	FP： not actually affected, but it is recognized as a disease
**Specificity**	$\frac{TN}{FP+TN}$	FN: actual illness, but was identified as being unaffected

Table S11. Model performance statistic.

	LR	SVM	Naive-Bayes	Ensemble
**Accuracy**	0.612500±0.074789	0.518750±0.035013	0.497917±0.058101	0.789583±0.108943
**Precision**	0.660069±0.155874	0.527778±0.139916	0.565625±0.135964	0.765104±0.141375
**Sensitivity**	0.631944±0.160185	0.621528±0.167532	0.729167±0.127047	0.87500±0.105263
**Specificity**	0.656250±0.227961	0.510417±0.252522	0.500000±0.252632	0.770833±0.178509

2 Supplementary Note

2.1 SAH intervention experiment

The mouse endovascular perforation model of SAH was induced as reported previously(Yujie et al., 2015;Amp and Wilkins, 2017). Briefly, mice were anesthetized with isoflurane. A sharpened 5-0 monofilament nylon suture was inserted rostrally into the left internal carotid artery from the external carotid artery stump and perforated the bifurcation of the anterior and middle cerebral arteries. Sham-operated mice underwent the same procedure without puncturing the artery. Tissues of white matters were taken for follow-up detection on day 3 after SAH.

The processed datasets for this study can be found in the github:

https://github.com/charlotte5683/SAH.git

ArrayExpress accession: E-MTAB-8407

https://www.ebi.ac.uk/arrayexpress/experiments/E-MTAB-8407/

2.2 LCN2 intervention experiment

According to methods described previously (Zuo et al., 2017), an intracerebroventricular injection was performed. Put simply, mice were placed on a stereotaxic apparatus (Rwdmall, Guangzhou, China) after anesthetized with 2% pentobarbital sodium (50 mg/kg, intraperitoneal). The bregma point was

then exposed and a small bone window was drilled into the bone of the left hemisphere. Then, 2 μL specific siRNAs was delivered into the lateral ventricle with a Hamilton syringe (Hamilton Company, Reno, NV, USA). The injection was performed 48 h before SAH. Tissues of white matters were taken for follow-up detection on day 1 and day 3 after SAH respectively.

The processed datasets for this study can be found in the github:

https://github.com/charlotte5683/LCN2.git

NCBI SRA accession: PRJNA575372

https://www.ncbi.nlm.nih.gov/sra/PRJNA575372

2.3 Code availability

Code used for predictive model is available at https://github.com/charlotte5683/SAH-code .

3 Reference

Amp, L.W., and Wilkins (2017). Correction to: Role of Periostin in Early Brain Injury After Subarachnoid Hemorrhage in Mice. *Stroke* 48**,** 1108-1111.

Yujie, C., Yang, Z., Junjia, T., Fei, L., Qin, H., Chunxia, L., Jiping, T., Hua, F., and Zhang, J.H. (2015). Norrin protected blood-brain barrier via frizzled-4/β-catenin pathway after subarachnoid hemorrhage in rats. *Stroke* 46**,** e91.

Zuo, S., Ge, H., Li, Q., Zhang, X., Hu, R., Hu, S., Liu, X., Zhang, J.H., Chen, Y., and Feng, H. (2017). Artesunate Protected Blood–Brain Barrier via Sphingosine 1 Phosphate Receptor 1/Phosphatidylinositol 3 Kinase Pathway After Subarachnoid Hemorrhage in Rats. *Molecular Neurobiology* 54**,** 1213-1228.
